# Stereopsis, Visuospatial Ability, and Virtual Reality in Anatomy Learning

**DOI:** 10.1155/2017/1493135

**Published:** 2017-06-01

**Authors:** Jan-Maarten Luursema, Marc Vorstenbosch, Jan Kooloos

**Affiliations:** Department of Anatomy, Radboud University Medical Center, 6525 GA Nijmegen, Netherlands

## Abstract

A new wave of virtual reality headsets has become available. A potential benefit for the study of human anatomy is the reintroduction of stereopsis and absolute size. We report a randomized controlled trial to assess the contribution of stereopsis to anatomy learning, for students of different visuospatial ability. Sixty-three participants engaged in a one-hour session including a study phase and posttest. One group studied 3D models of the anatomy of the deep neck in full stereoptic virtual reality; one group studied those structures in virtual reality without stereoptic depth. The control group experienced an unrelated virtual reality environment. A post hoc questionnaire explored cognitive load and problem solving strategies of the participants. We found no effect of condition on learning. Visuospatial ability however did impact correct answers at *F*(1) = 5.63 and *p* = .02. No evidence was found for an impact of cognitive load on performance. Possibly, participants were able to solve the posttest items based on visuospatial information contained in the test items themselves. Additionally, the virtual anatomy may have been complex enough to discourage memory based strategies. It is important to control the amount of visuospatial information present in test items.

## 1. Introduction

### 1.1. Virtual Reality for Anatomy Learning

Virtual reality (VR) aims to provide its users with an illusory environment by completely replacing direct sensory stimuli for artificially generated (or mediated) sensory stimuli. Complete immersion in a virtual environment would enable interaction with virtual objects similar to interaction with real objects [1]. In addition to this, VR allows the development of interaction modalities for which no real-world analog exists. For the study of human anatomy, this promises extraordinary versatility and flexibility in the presentation and exploration of anatomical objects, at a fraction of the cost of maintaining dissection facilities [2–4]. However, full VR is technologically challenging and has not yet been implemented. The ongoing development of virtual reality is reflected in the lack of consensus in the literature as to the effectiveness of digital 3D representations for human anatomy learning.

Older studies often report ambiguous or negative findings (e.g., [5, 6]). These studies however do not utilize the full potential of virtual reality, using 3D computer models in a “desktop VR” setting. In desktop VR, sources of spatial information such as physical size and tactile/force feedback are lost, and there is no direct interaction or sense of sharing space with virtual anatomical objects. Navigating such an environment might add to the cognitive load of the participants experienced during those experiments, which in turn could have biased the results of those studies. This is corroborated by studies working with a more complete implementation of virtual reality principles, which report a positive contribution of virtual reality for anatomy learning (the study of [2] provides a review and meta-analysis; [7, 8]). Despite uncertainty as to its effectiveness, virtual human anatomy has become an integral part of the medical curriculum [9].

### 1.2. Stereopsis in Digital Anatomy Learning

Besides the loss of tactile and force feedback, desktop VR anatomy comes with another limitation: the spatial information provided by the visual depth cue of* stereopsis* is also lost. Stereopsis refers to the experience of spatial depth based on the brain's comparison of synchronous and overlapping visual information provided by an organism's paired eyes. Stereopsis is one of the most important depth cues for estimating distance and size for objects in close proximity to oneself ([10]; see also [11]). The loss of spatial information caused by digital mediation makes it harder to interpret spatially complex objects or contexts [12], especially for people of low visuospatial ability [13]. Human anatomy is spatially complex, and people of high visuospatial ability have an advantage in anatomy learning ([14] provides a review).

The visual depth cue of stereopsis however can be implemented in digital learning environments. Luursema et al. [7] found that learning in a digital environment which implemented both stereopsis and direct manipulation allowed students of low visuospatial ability to perform on par with their high visuospatial counterparts in visual tests of anatomical knowledge. So far, this only has been shown in experiments with psychology students who studied anatomical material of relatively low complexity. To provide ecological validity to these earlier results, we aim to verify the conclusions of these studies working with medical students and more complex digital anatomical material.

### 1.3. A New Wave of Virtual Reality Devices

One way to implement stereopsis digitally is to use a virtual reality setup. A new wave of affordable virtual reality head-mounted devices has come to market at the time of writing, including the Oculus Rift, Google cardboard, and Samsung's Gear VR (http://www.wired.co.uk/magazine/archive/2015/07/features/vr-total-immersion). With these devices, a long standing interest in the use of virtual reality for anatomy learning has gained new momentum (e.g., http://anatomy.stanford.edu/research---development.html). Although immersion in a virtual reality environment can lead to a heightened sense of presence, object manipulation in such an environment is not intuitive and may add to the users' cognitive load [15]. This might especially be an issue for students of low visuospatial ability, who are more likely to depend on reasoning strategies for solving all task aspects, in contrast to people of high visuospatial ability, who can bring spatial visualization strategies to bear when solving visuospatial task aspects [16]. We aim to (1) explore the contribution of stereopsis to digital anatomy learning and to (2) assess the usefulness of virtual reality for anatomical learning for users of differing visuospatial ability.

### 1.4. The Experiment

**Figure 1:**
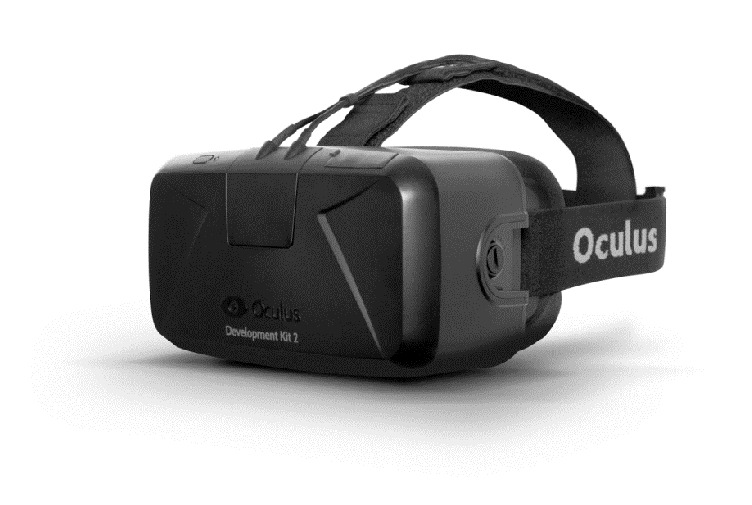
Oculus Rift SDK 2. The virtual learning environment used in this study was developed for this head-mounted display.

To this end, we created a virtual anatomy learning environment to study the anatomy of the neck. In a three-condition randomized experiment, participants were tested for anatomical spatial knowledge of the C1 and C2 cervical vertebrae after either studying this anatomy in a stereoptic condition, in a nonstereoptic condition, or exploring an unrelated virtual environment. A virtual reality head-mounted display, the Oculus Rift SDK 2 (Figure 1), was used for all conditions of the study phase. All participants were tested for stereoptic vision and visuospatial ability. We hypothesized that both virtual anatomy groups would outperform the nonanatomical control condition. We also expected the participants in the stereoptic condition would outperform the participants of the nonstereoptic condition, mostly caused by increased learning in participants of low visuospatial ability [7].

To assess spatial anatomical knowledge, we created a test that asks the participant to localize a cross section of the studied anatomy on a frontal view of that same anatomy. The student can do this by clicking one of a number of horizontal lines drawn over this frontal view. This minimizes reliance on other aspects of anatomical knowledge (e.g., verbal, functional), while at the same time being relevant for learning to apply anatomical knowledge in a clinical setting, for example, to interpret cross-sectional material resulting from radiological or histological imaging.

To clarify the outcomes of this experiment and to inform future research, we distributed a questionnaire about cognitive load and visuospatial versus analytical problem solving strategy among all participants after the experiment. We reasoned that the virtual reality environment of the study phase might have added extraneous cognitive load, caused by novelty and the not entirely intuitive interaction with this environment. We hypothesized less cognitive load would be experienced by participants of high visuospatial ability, because they were likely to combine visuospatial strategies with analytical strategies. In contrast participants of low visuospatial ability were thought to depend on analytical strategies alone, both for visuospatial and for nonvisuospatial task aspects, which would have led to a comparatively high cognitive load.

## 2. Materials and Methods

### 2.1. Participants

Eighty first-year medical and biomedical students of the Radboud University Medical Center (Nijmegen, Netherlands) signed up for participation in this study and were randomly distributed over three conditions. Sixteen students did not follow up, and data for one participant who did not see stereoptically was excluded from analysis (see below). Thus, data for 63 participants were analyzed. The participants were distributed as follows over the three experimental conditions: 22 students (8 male) participated in the stereoptic condition, 23 (7 male) in the nonstereoptic condition, and 18 (10 male) in the control condition. Mean age was 19.3, ranging between 18 and 25. All participants voluntarily signed an informed consent document. Participants received a 15-Euro gift voucher for their participation. No formal ethics review was sought, as this is not required for this type of study under Dutch law.

### 2.2. Study Protocol

The following steps were part of each session. They are listed in the same order as offered during the experiment. Total time for each session was about 60 minutes:* Welcome*, informed consent, description and explanation of the session, and opportunity for the participant to ask questions.

During the* pretest*, we provided the participants with four example questions so they could use the study phase to prepare for the similar questions of the posttest.

The* study phase* differed for each experimental condition, except for duration, which was 150 seconds. This duration was based on previous research [17] and a three-person pilot. We found that around the two-minute mark participants start losing focus, which led us to believe a study phase exceeding 150 seconds would not lead to more learning. Participants were alerted to the time left for exploring the study phase environment at the thirty-second, one-minute, and two-minute mark. Students were randomly distributed over the following three conditions.

**Figure 2:**
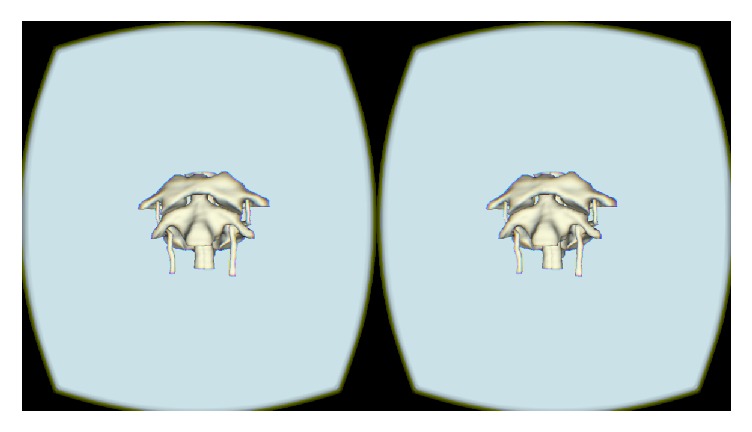
View of the learning environment the participants interacted with during the study phase. In this screenshot the 3D anatomy looks a bit small. This is caused by the relatively large screen area reserved within the Oculus Rift for peripheral vision and does not correspond to the user experience.

*(1) The Stereoptic Condition*. Participants in this group studied a 3D reconstruction of anatomical objects of the deep neck wearing the Oculus Rift SDK 2 head-mounted display (Figure 2). The anatomical objects were offered stereoptically in this condition, meaning that each eye was presented with a slightly different view of the virtual objects, to enable the brain to generate the experience of stereoptic visual depth. The virtual anatomy was positioned at a set distance from the participants' head, and they could rotate the objects using the arrow keys of a regular keyboard. 

*(2) The Nonstereoptic Condition*. This condition was identical to the stereoptic condition, with the exception of the virtual objects being offered such that both eyes were presented with the exact same visual perspective. The visuospatial depth cue of stereopsis was thus not available to the participants in this condition. 

*(3) The Control Condition*. Participants in this condition did wear the Oculus Rift headset for 150 seconds and only got to explore a virtual sea world instead of test-related human anatomy.

**Figure 3:**
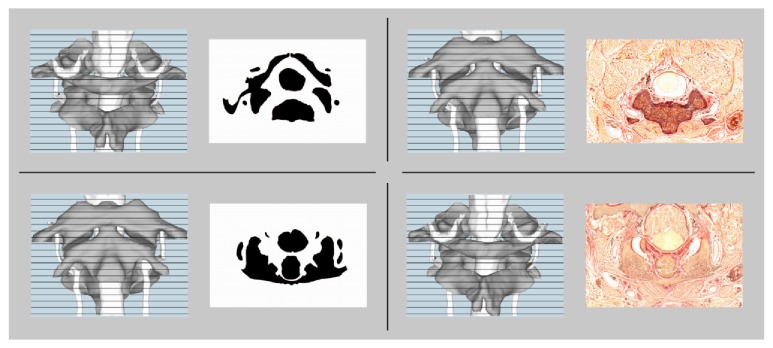
Four example questions of the posttest. The participants were instructed to click the horizontal line over the left-hand picture corresponding to the cross section shown on the right.

The anatomy of the neck was selected for reasons of complexity and curriculum. The selected structures were spatially complex but with enough salient characteristics to allow participants to uniquely localize transverse cross sections along the transverse axis of a frontal view (Figure 3). The experiment was scheduled to allow the participation of first-year medical students experienced in studying academic anatomy, but naive to this specific region (the study of which was lined up next in the curriculum).

In order to minimize a potential speed-accuracy trade-off during the* posttest*, students were instructed to prioritize working carefully, but also to work fast. In each of the 43 questions of the posttest, participants were presented on the left side of the screen with either a ventral view or a dorsal view of the anatomy just studied, overlaid with horizontal, clickable lines. In each consecutive question, to the right a different cross section of the studied anatomy was displayed. This could either be a picture of the actual cross section on which the 3D reconstruction they studied was based, or an abstracted version of such a cross section (Figure 3). We used these two variants to implement different levels of complexity (the abstracted version was easier to interpret). The participants' task was to click the horizontal line over the left-hand picture that corresponded with the cross section shown to the right. If a participant indicated a line above or below the official correct answer as correct, this was accepted given the similarity between many consecutive cross sections. Number of correct answers and average response times were collected.

*Visuospatial Ability Test*. To assess participants' visuospatial ability, we used a test battery consisting of electronic versions of two standard tests in this area. Both tests assess the ability to mentally manipulate relatively complex visuospatial stimuli; they were Vandenberg and Kuse's Mental Rotation Test [18, 19] and the Surface Development test [20]. 

*Anatomical Knowledge Test*. To control for preexisting individual differences in anatomical (nonspatial) knowledge of the area under study, a 10-item multiple choice questionnaire was filled out digitally by all participants. This questionnaire was based on a consensus discussion of two expert anatomists (authors JK and MV) who compared and edited question lists they had previously compiled independently.

*Stereopsis Test*. Some 5 to 10 percent of a population does not perceive stereoptic visual depth, often due to early astigmatism [21]. Not being able to see stereoptically is an exclusion criterion for this study, so we tested all participants for stereoptic vision using the Random Dot 3 LEA SYMBOLS® Stereoacuity Test [22], a standard test in this area. Performance data for one participant were excluded from analysis based on this test.

A* demographics questionnaire* was filled out by all participants digitally at the end of the session.

The link to a digital, 12-item 5-point Likert scale,* post hoc mixed cognitive load/problem solving strategy questionnaire* was emailed to the participants five months after the experiment. This was done to contextualize our research findings and inform future research designs. Four of the questions assessed cognitive load (adapted from [23]), four assessed the use of analytic problem solving strategies, and four questions assessed the use of visuospatial problem solving strategies (Appendix).

### 2.3. Materials

The experiment was run from a desktop computer under Windows 7 professional, SP1. The computer ran on an Intel Xeon W3565 CPU @ 3.20 Ghz, with 6-gigabyte RAM. Video was provided by a Nvidia Quadro 5000 video card. The Oculus Rift SDK2 VR head-mounted display was used to explore the virtual anatomical learning environment during the study phase.

The study phase was created using the Unity game engine v. 5.0, with additional Oculus Rift plugins (available at https://developer.oculus.com/downloads/). Pretest and posttest were made and run in Open Sesame v. 2.9, a package for creating psychological experiments, provided as open source to the community by the Vrije Universiteit Amsterdam [24]. Analysis was done in the SPSS statistical package, v. 21. The anatomical knowledge questionnaire and cognitive load/problem solving strategy questionnaire were made in LimeSurvey v. 1.92, the demographics questionnaire in Microsoft Excel 2013.

The virtual anatomical objects were based on a manual segmentation of histological coupes of the neck. These histological coupes also formed the basis for the slices used at the pretest and posttest stage. For the graphical aspects of this, the Gimp v. 2.8 and Inkscape v. 0.91 were used, and the 3D reconstructions were made with the Surfdriver package and Meshlab v. 1.3.4.

The post hoc questionnaire was created in the LimeSurvey v. 1.92 package for creating web-based surveys.

### 2.4. Data Preparation/Analysis

#### 2.4.1. Experimental Analysis

Performance at the anatomical knowledge test was at chance level. Of the ten multiple choice questions, four had four answering options, and six had three answering options. Overall, for participants this results in 30 percent (3 answers) correct as performance at chance level. Answers came in at a mean of 2.8 correct, with an SD of 1.3, a minimum of 0, and a maximum of 6 answers correct. Consequently, this variable was not further analyzed in relation to other variables. This however rules out preexisting anatomical knowledge as a source of variability on the experimental results.

The scores of the two visuospatial ability subtests were normalized and mean values from both were calculated as a proxy for participants' visuospatial ability. For each participant, the number of correct posttest answers and the average posttest question reaction time were calculated. The participants performed above chance, and a KS1 test showed these variables to not deviate significantly from the normal distribution, allowing for analysis of variance testing. We also calculated correlations between correct answers and reaction times to assess a potential speed-accuracy trade-off.

An ANOVA for visuospatial ability × experimental condition was performed to verify a similar distribution of visuospatial ability between experimental groups. To assess the effect of stereoptic vision on anatomical learning in a virtual reality environment, an ANCOVA for the impact of experimental condition on correct answers and reaction times was performed, with spatial ability as a covariable. Additional ANOVAs were done to compare the effects of the three conditions pair-wise.

#### 2.4.2. Post Hoc Questionnaire Analysis

Responses from 51 of the 63 participants were collected. The responses for the four items of each of the three question categories were averaged, resulting in variables for cognitive load, visuospatial problem solving strategy, and analytic problem solving strategy. A strategy preference variable was created by calculating the difference values of the visuospatial problem solving strategy and analytic problem solving strategy responses.

To contextualize our main results, correlations were calculated for cognitive load, strategy preference, and visuospatial ability × posttest correct answers and posttest reaction times.

## 3. Results

### 3.1. Experiment

Descriptive statistics of the main variables, split by experimental condition, are provided in Table 1. A moderate but significant correlation was found between correct answers and reaction times (*r* = .36, *p* < .005), which means that participants who took more time during the posttest phase were more likely to provide correct answers. This speed-accuracy trade-off did not influence the experimental results as explained below.

The ANOVA for visuospatial ability × experimental condition showed a similar distribution of visuospatial ability over the experimental conditions, indicating a random selection from a homogenous population for each condition.

The ANCOVA to assess the influence of digitally implemented stereopsis on virtual learning showed no effect of experimental condition on either reaction times or correct answers. As a covariate, visuospatial ability did impact correct answers (but not reaction times), at *F*(1) = 5.63 and *p* = .02 (Table 2). Further comparing the experimental conditions and the control condition in paired ANOVAs, we did not find a difference between the two experimental conditions, and we also did not find a difference between experimental condition and the control condition. All of this renders the potential effect of a speed-accuracy trade-off mentioned above a moot point. Given these results, no further interaction effects for visuospatial ability and condition were studied as they would be statistically meaningless.

### 3.2. Post Hoc Questionnaire

Neither cognitive load nor strategy preference correlated significantly with posttest correct answers or visuospatial ability. Strategy preference, but not cognitive load, did correlate with posttest reaction times, *r* = −.40 and *p* < .01. This indicates that the people who reported both a high use of analytical problem solving strategies and a low use of visuospatial strategies performed faster.

## 4. Discussion

Confirming earlier research in this area, visuospatial ability positively impacted anatomical learning [7, 14]. In contrast, we were not able to confirm earlier research that suggests stereopsis in digital learning environments can positively influence learning [17]. We did in fact not find an effect for either of our experimental conditions compared to each other, or to the control condition.

Possibly, regardless of whether any learning took place during the study phase, participants were able to solve the questions of the posttest based solely on the visual information in these questions, and this might have been a more attractive strategy than retrieving the studied anatomy from memory. If this is in fact the reason for our (lack of) results, we may conclude that in both future studies and educational practice it will be extremely important to better control the amount of visuospatial information available in test questions, especially when we start developing tests that rely more heavily on spatial reasoning compared to traditional, mostly text oriented tests [25, 26]. We had no reason to expect such an effect however, given the experience of one of the authors (JML) who in an earlier series of studies with a very similar design* did* find effects for different study phase conditions [7, 17]. These contrasting results may have been caused by the earlier studies working with participants naive to the study of human anatomy, who might have been less able to use visuospatial information available in anatomical questions compared to medical students.

An additional explanation for the lack of effect of our experimental treatment is that the pretest questions may have primed our participants to pay little attention to the study phase. Finding they would be able at posttest to answer the questions based on the information available in the questions themselves, they may have interpreted the study phase as an appropriate time for relaxation.

In contrast to received wisdom that visuospatial problem solving strategies temporally outperform analytic strategies for visuospatial problems [27], participants reporting a relative high use of analytical problem solving strategies performed faster. The high complexity of the virtual anatomy may have caused visuospatial problem solving strategies to actually slow participants down. If this is so, there might be an optimum level of visuospatial complexity where high visuospatial people outperform low visuospatial people, above which low visuospatial people, used to revert to analytical strategies sooner, will actually start outperforming their high visuospatial counterparts again. More studies are needed that explore the relation between visuospatial ability, reasoning ability, problem solving strategies, and visuospatial complexity.

### 4.1. Limitations

The implementation of virtual reality was incomplete. For example, exploration during the study phase was somewhat cumbersome: the objects could only be rotated using the arrow keys of the keyboard. A motion controlled sensor could have made interaction more natural; however this technology was not available to us when we were building the experiment. Secondly, the virtual anatomical objects were not positioned in a specific location in virtual space; instead they were tethered to the Oculus Rift at a set distance and orientation. While this allowed us to abolish “distance to the objects” as a source of variability between participants, this also distorted the illusion of being in a stable environment containing virtual objects. In a follow-up study, we will explore gesture recognition technology (such as the Leap or the Kinekt) to implement interaction and provide the virtual objects with a stable location.

Cognitive load was not found to influence any of our results; however high stimulus complexity and unfamiliarity with the virtual reality hardware might still have hindered learning during the study phase. Easier digital anatomical objects might have led to more learning during the study phase, which could have translated to participants using visual memory based strategies more often during posttest execution. More variety in terms of visuospatial complexity for the objects studied will allow us to test this hypothesis. In addition to this, a familiarization phase at the beginning of the experiment for the virtual reality setup should diminish extraneous cognitive load caused by unfamiliarity with this type of technology.

### 4.2. Future Work

In a follow-up study in preparation we want to explore the relation between visuospatial ability, spatial complexity, and problem solving strategy. We will prime our participants to the existence of both visuospatial and reasoning problem solving strategies to enable them to report their use of those throughout the experiment. We will also systematically vary the complexity of our stimulus materials. Thirdly, we will minimize the amount of visuospatial information present in posttest questions to stimulate recall of information learned during the study phase. Lastly, we will continue developing our virtual reality environment to maximize the feeling of being present by fully localizing the virtual objects and by implementing easier to use interaction technology.

## 5. Conclusions

We do not know yet whether stereopsis in digital learning environments helps or hinders anatomy learning. It is important in both research and educational practice to control the amount of visual information provided by test questions. Given the speed of the development of virtual reality enabling technologies, research into the use of VR for anatomical learning is a moving target.

## Figures and Tables

**Table 1:** Descriptive statistics of the main variables, split by experimental condition. Reported as mean (standard deviation).

Variables∖conditions	Stereoptic	Nonstereoptic	Control
Posttest accuracy (# correct from 43 items)	18.86 (5.65)	18.78 (5.47)	18.61 (4.47)
Posttest speed (*s*)	22.39 (12.19)	22.43 (10.48)	23.15 (15.95)
Visuospatial ability (normalized)	−0.12 (1.18)	0.03 (0.99)	0.11 (0.78)
Strategy bias (difference score from 2 Likert 5 pt. scale variables)	0.15 (0.48)	0.15 (0.68)	−0.20 (1.15)
Cognitive load (5 pt. Likert)	3.01 (0.53)	2.93 (0.55)	2.77 (0.53)

**Table 2:** Results of our ANCOVA, investigating the relation between posttest performance, experimental condition, and visuospatial ability.

Source	Dependent variable	*F* (df)	*p*
Experimental condition	Correct answers	.07 (2)	.93
Experimental condition	Total duration	.01 (2)	.99
Visuospatial ability	Correct answers	5.63 (1)	.02
Visuospatial ability	Total duration	.80 (1)	.37

## Appendix

### A. Post Hoc Questionnaire “Cognitive Load and Problem Solving Strategies” (5-Point Likert Scale, Translated from Dutch)

#### A.1. Cognitive Load

- The anatomical objects I had to study were very complex.
- The virtual reality setup was very hard to use.
- The explanation of the experiment was very unclear.
- After the study phase my understanding of the anatomy of the topmost cervical vertebrae was much better.

#### A.2. Problem Solving Strategy, Analytic

- I compared specific elements of the test's cross sections with specific elements of the frontal or dorsal view.
- I systematically discarded options until the correct answer remained.
- I always combined the top and bottom elements of the cross sections.
- I rarely recalled the studied model in order to solve the test questions.

#### A.3. Problem Solving Strategy, Visuospatial

- I mentally rotated the cross section to see where it would fit in the frontal or dorsal view.
- It was easy for me to visually recall the studied anatomy to help me identify the cross sections' correct location.
- It was easy for me to mentally rotate the studied anatomy help me identify the cross sections' correct location.
- The negative spaces (“holes”) in the cross sections really helped me select the correct answer.
